# Cardiohepatic Syndrome Presenting as Large-Volume Ascites in Acute Decompensated Heart Failure: A Case Report

**DOI:** 10.7759/cureus.113336

**Published:** 2026-07-24

**Authors:** Brendan Masi, Anvitha Kambham, Abraham E Libman, Himanshukumar Nayak, Roxana Lazarescu

**Affiliations:** 1 Medicine, Touro College of Osteopathic Medicine, New York, USA; 2 Internal Medicine, Touro College of Osteopathic Medicine, New York, USA; 3 Internal Medicine, Wyckoff Heights Medical Center, New York, USA

**Keywords:** cardiac ascites, cardiohepatic syndrome, congestive hepatopathy, diastolic dysfunction, heart failure with preserved ejection fraction (hfpef), hypervolemia, left ventricular hypertrophy, right heart failure, systemic venous congestion, venous congestion

## Abstract

Congestive hepatopathy is an underrecognized manifestation of elevated cardiac filling pressures and systemic venous congestion in heart failure. Although acute decompensated heart failure often presents with pulmonary edema and respiratory compromise, advanced diastolic dysfunction may also produce hepatic congestion, ascites, pleural effusions, and cardiorenal syndrome. We present the case of a 48-year-old male with hypertension, hyperlipidemia, type 2 diabetes mellitus, and recently diagnosed heart failure with preserved ejection fraction (HFpEF) who presented with progressive dyspnea, orthopnea, bilateral lower extremity edema, and declining exercise tolerance. Laboratory evaluation demonstrated hypervolemic hyponatremia, acute kidney injury, hyperbilirubinemia, and cholestatic-predominant liver injury. Echocardiography revealed preserved ejection fraction with severe concentric left ventricular hypertrophy and findings suggestive of elevated right-sided filling pressures, including a dilated inferior vena cava with reduced inspiratory collapse. Left heart catheterization demonstrated significantly elevated left ventricular end-diastolic pressure despite nonobstructive coronary artery disease. Abdominal imaging showed hepatomegaly, ascites, mesenteric congestion, pleural effusion, and gallbladder wall thickening without biliary obstruction. The patient improved with aggressive intravenous diuresis and guideline-directed therapy. The combined clinical, laboratory, imaging, hemodynamic, and treatment-response findings were most consistent with congestive hepatopathy and cardiac ascites due to severe systemic venous congestion. Preserved systolic function does not exclude severe extracardiac congestion, including hepatic congestion, ascites, and cardiorenal syndrome. Recognizing congestive hepatopathy can prevent unnecessary hepatobiliary evaluation and direct appropriate hemodynamic management and decongestion.

## Introduction

Heart failure with preserved ejection fraction (HFpEF) is a prevalent cause of hospitalization and multiorgan dysfunction and remains a major contributor to cardiovascular morbidity worldwide [1]. Clinical manifestations most commonly include dyspnea, pulmonary edema, and exercise intolerance related to elevated cardiac filling pressures and impaired diastolic relaxation. In advanced HFpEF, sustained elevation of left-sided filling pressures may lead to postcapillary pulmonary hypertension, increased right ventricular afterload, and, over time, right ventricular dysfunction. Rising right-sided filling pressures can then transmit congestion to the systemic venous circulation [1,2]. However, chronic systemic venous congestion may also produce substantial extracardiac manifestations, including congestive hepatopathy, cardiac ascites, pleural effusions, mesenteric congestion, and cardiorenal syndrome [2].

Congestive hepatopathy results from prolonged passive hepatic venous congestion secondary to elevated right-sided cardiac pressures [3]. Chronic hepatic congestion may produce cholestatic liver injury, hepatomegaly, hyperbilirubinemia, ascites, and, in advanced cases, fibrosis and cardiac cirrhosis. In patients with preserved left ventricular systolic function, diagnosis can be challenging because these findings resemble primary hepatobiliary pathology [4].

Cardiac ascites is another manifestation of advanced heart failure and elevated venous pressures. When possible, performing a diagnostic paracentesis should demonstrate a high serum-ascites albumin gradient (SAAG) and elevated ascitic protein concentration [5]. This helps to distinguish cardiac ascites from cirrhotic etiologies. In addition, invasive hemodynamic assessment through right heart catheterization can help to clarify volume status and guide therapy in complex presentations [6].

We present a case of severe systemic venous congestion in a relatively young patient with HFpEF who developed congestive hepatopathy, ascites, pleural effusion, hyponatremia, and cardiorenal syndrome despite having preserved systolic function with nonobstructive coronary disease.

## Case presentation

A 48-year-old male with a past medical history of hypertension, hyperlipidemia, type 2 diabetes mellitus, and recently diagnosed HFpEF presented to the emergency department with progressive shortness of breath, bilateral lower extremity edema, orthopnea, and worsening exercise tolerance. He was recently hospitalized approximately one month earlier for decompensated diastolic heart failure. Complete admission vital signs could not be confirmed from the available records.

On presentation, the patient reported being unable to walk more than one block without stopping because of dyspnea. He also reported requiring three pillows at night because he was unable to comfortably lie down flat/supine. He denied chest pain, palpitations, fever, abdominal pain, or infectious symptoms.

Initial chest radiography demonstrated no focal infiltrates. Pertinent laboratory values during hospitalization are summarized in Table 1. Arterial blood gas analysis showed mixed hypoxemic hypercapnic respiratory failure, prompting initiation of bilevel positive airway pressure (BiPAP), after which his respiratory status improved. Brain natriuretic peptide (BNP) was elevated on admission compared with the prior admission and increased further during hospitalization.

**Table 1:** Pertinent laboratory findings during hospitalization. A dash indicates that a value was unavailable or not reported. Ranges represent values documented during the specified interval. BNP, brain natriuretic peptide; AST, aspartate aminotransferase; ALT, alanine aminotransferase

Parameter	Baseline / prior	Admission / initial	Peak / hospital course	Reference range
pH	—	7.17	—	7.35–7.45
pCO₂ (mmHg)	—	59	—	35–45
pO₂ (mmHg)	—	43	—	80–100
Bicarbonate (mmol/L)	—	21.5	—	22–26
BNP (pg/mL)	1568	2013	>3300	<100
Sodium (mmol/L)	—	127–128	127–128	135–145
Creatinine (mg/dL)	~0.8	1.62	1.62	0.7–1.3
Troponin (ng/L)	—	286	302	<20 (institutional reference range)
Alkaline phosphatase (U/L)	—	—	280	40–129
Total bilirubin (mg/dL)	—	—	3.0	0.2–1.2
AST (U/L)	—	—	130	10–40
ALT (U/L)	—	—	173	7–56

Physical examination was notable for bilateral pulmonary crackles and lower extremity pitting edema extending to the knees. Laboratory evaluation revealed hyponatremia, acute kidney injury with increased creatinine from baseline, and elevated blood urea nitrogen. Troponin levels were mildly elevated with a small interval rise. This mild elevation was interpreted as demand ischemia in the setting of acute decompensated heart failure, given that the patient denied chest pain, and his prior coronary angiography demonstrated nonobstructive coronary disease.

Liver function testing demonstrated mixed cholestatic liver injury, with elevated alkaline phosphatase, hyperbilirubinemia, elevated direct bilirubin, and elevated aminotransferases. Viral hepatitis serologies were negative.

Transthoracic echocardiography showed preserved left ventricular systolic function with ejection fraction of 55-60%, severe concentric left ventricular hypertrophy, mildly elevated right ventricular systolic pressure (~35 mmHg), and a dilated inferior vena cava with reduced inspiratory collapse. These findings are suggestive of elevated right-sided filling pressures. Left heart catheterization revealed nonobstructive coronary artery disease with only 30% mid-left anterior descending stenosis and iFR of 0.97. Left ventricular end-diastolic pressure, however, was significantly elevated at 29 mmHg. Given the degree of hypertrophy, despite reportedly controlled hypertension, cardiology raised concern for restrictive cardiomyopathy, including possible cardiac amyloidosis, and recommended outpatient cardiac magnetic resonance imaging (MRI).

**Figure 1:**
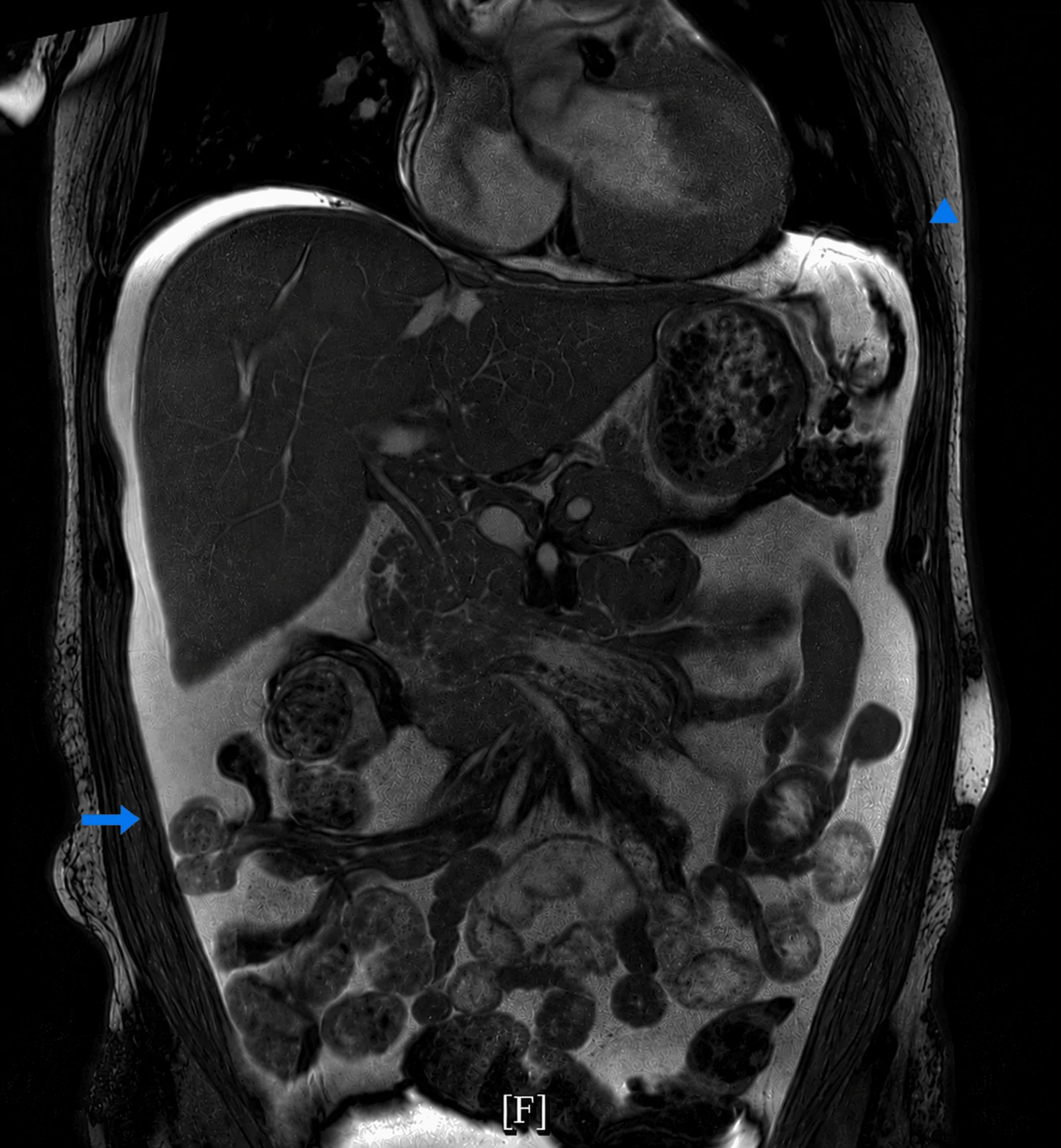
Coronal magnetic resonance cholangiopancreatography (MRCP) without contrast demonstrating large-volume ascites Coronal MRCP without contrast demonstrating large-volume ascites (arrow) and an associated right pleural effusion (arrowhead), consistent with systemic venous congestion. No biliary obstruction or pancreatic mass was identified.

Abdominal imaging revealed significant systemic venous congestion. Specifically, magnetic resonance cholangiopancreatography (MRCP) showed hepatomegaly, large-volume ascites, right pleural effusion, and gallbladder wall thickening without biliary obstruction (Figure 1).

**Figure 2:**
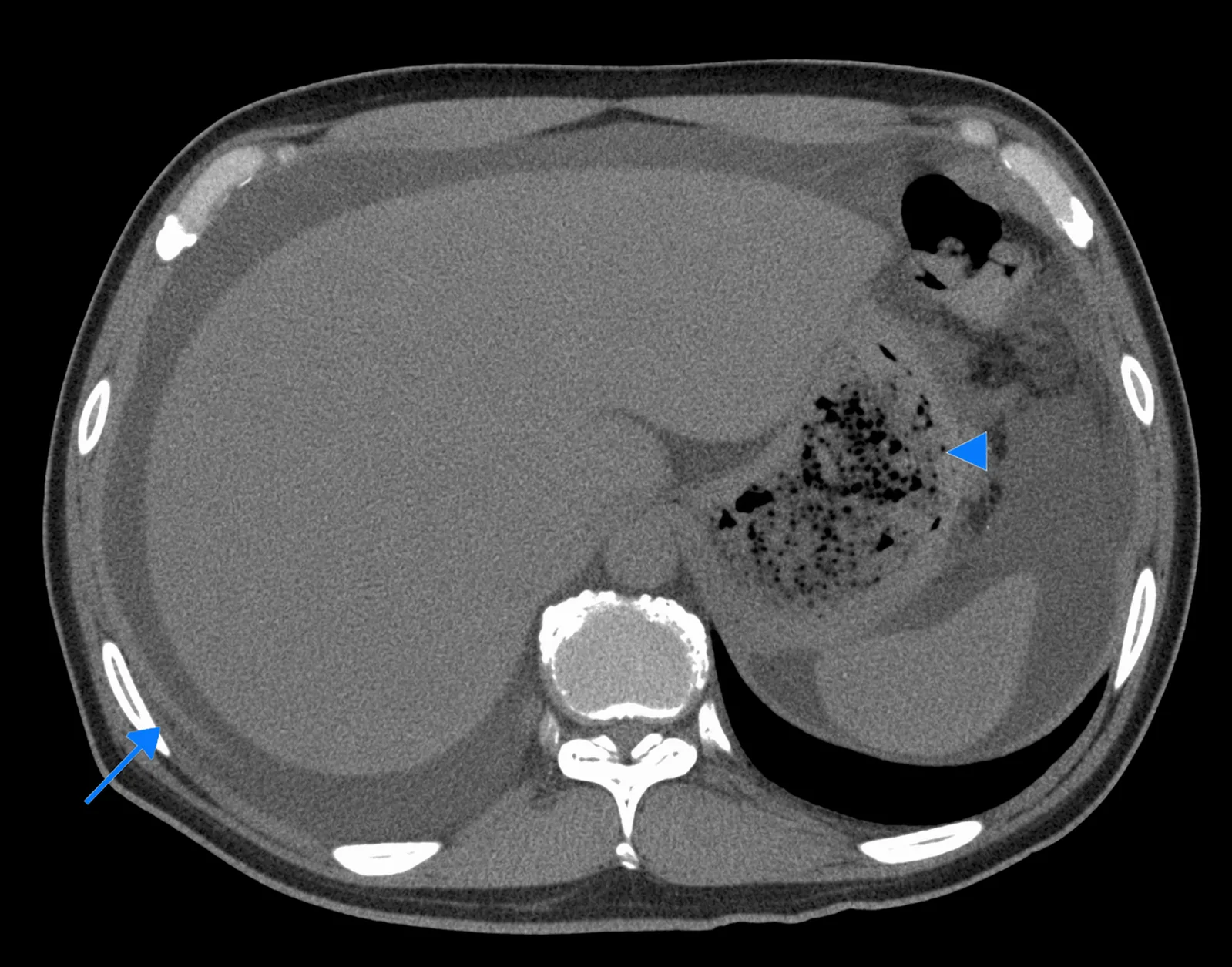
Axial computed tomography (CT) of the abdomen and pelvis without contrast demonstrating moderate abdominopelvic ascites Axial CT of the abdomen and pelvis without contrast demonstrating moderate abdominopelvic ascites (arrow) with diffuse mesenteric congestion (arrowhead), findings consistent with systemic venous congestion.

Computed tomography (CT) scan of the abdomen and pelvis showed mild-to-moderate ascites with generalized mesenteric congestion (Figure 2).

**Figure 3:**
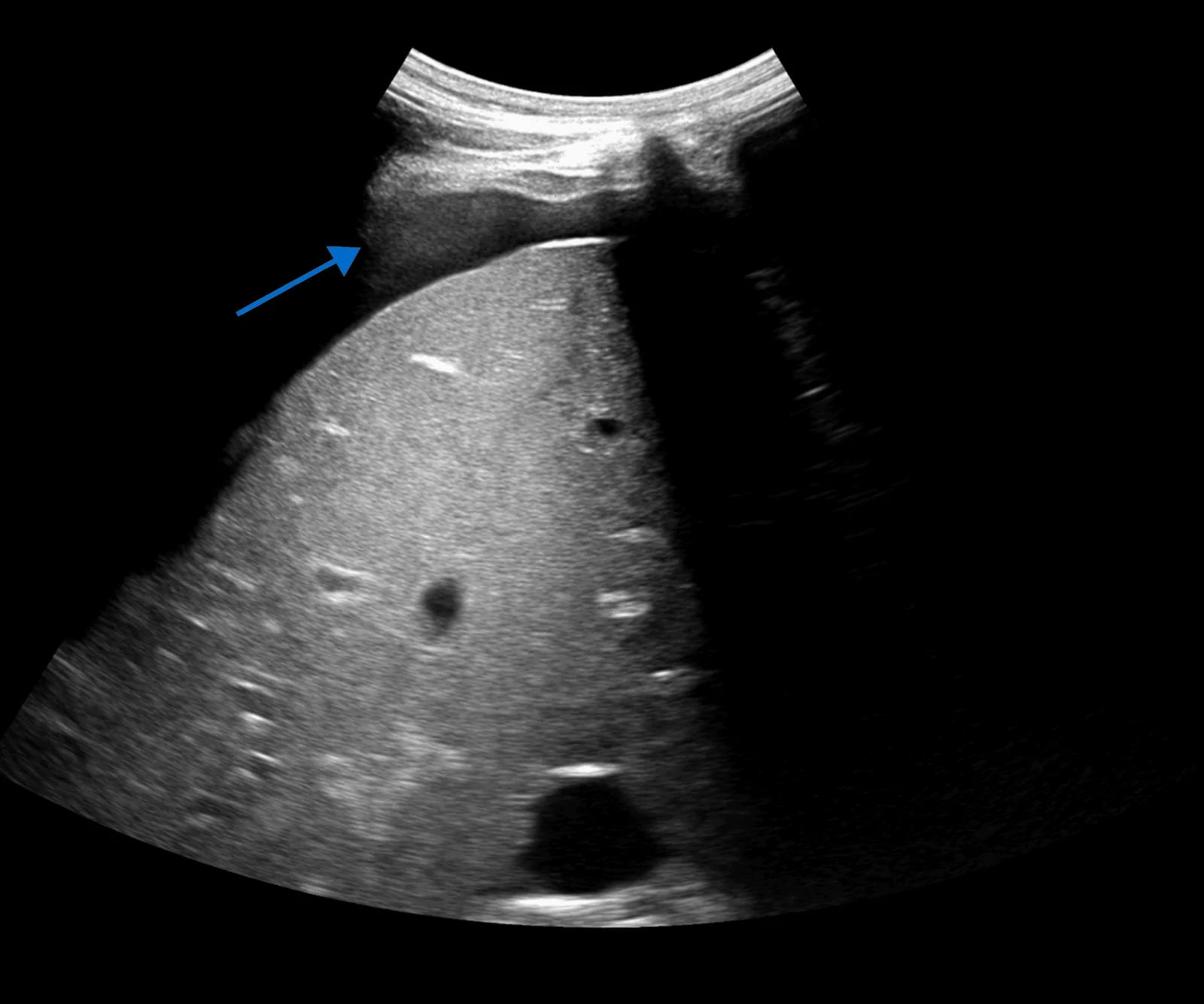
Ultrasonography (US) before planned diagnostic paracentesis demonstrating a thin residual perihepatic fluid collection US performed immediately before planned diagnostic paracentesis demonstrating a thin residual perihepatic fluid collection (arrow). Following aggressive diuresis, the volume of ascites had decreased substantially and was no longer sufficient for safe diagnostic paracentesis; the procedure was therefore deferred.

Gastroenterology and interventional radiology were then consulted for diagnostic paracentesis to further evaluate the ascites. By the time of ultrasound (US)-guided evaluation, however, insufficient drainable ascitic fluid remained, and the procedure was cancelled, likely reflecting a positive response to aggressive diuresis (Figure 3).

The patient was treated with intravenous furosemide in addition to empagliflozin and spironolactone. Lisinopril, carvedilol, atorvastatin, and metolazone were temporarily held during portions of hospitalization due to acute kidney injury, bradycardia, worsening hyponatremia, and transaminitis. Hyponatremia was managed with fluid restriction, sodium chloride supplementation, and Ure-Na therapy. Renal function and liver enzyme abnormalities gradually improved with decongestion, and the patient was discharged with outpatient cardiology follow-up and recommendation for further evaluation of possible restrictive cardiomyopathy.

## Discussion

Our patient developed severe systemic venous congestion with congestive hepatopathy and cardiac ascites despite preserved systolic function. Although acute decompensated heart failure classically presents with pulmonary edema and respiratory compromise, cases such as this show how advanced diastolic dysfunction may produce substantial extracardiac congestion, which can involve the liver, kidneys, mesentery, and pleural spaces.

The patient demonstrated multiple objective findings supporting elevated venous pressures. Specifically, he had bilateral edema, pleural effusion, ascites, hepatomegaly, mesenteric congestion, dilated IVC, and markedly elevated left ventricular end-diastolic pressure of 29 mmHg. Taken together, these findings strongly suggested severe systemic congestion despite the patient’s preserved ejection fraction.

Congestive hepatopathy results from chronic passive hepatic venous congestion caused by elevated right atrial and systemic venous pressures [2]. Elevated sinusoidal pressure impairs hepatic blood flow and oxygen diffusion, and this produces hepatocyte dysfunction and cholestatic injury [3]. Laboratory abnormalities often include hyperbilirubinemia and elevated alkaline phosphatase with comparatively mild transaminase elevation, as demonstrated in this patient. Similarly, imaging may show hepatomegaly, ascites, gallbladder wall thickening, and pleural effusions, just as we have seen here. Consequently, chronic untreated congestion may ultimately progress to fibrosis and cardiac cirrhosis [3].

The presence of ascites broadened the differential diagnosis and raised concern for primary hepatobiliary disease [4]. However, the overall clinical picture favored cardiac ascites resulting from systemic venous congestion. Diagnostic paracentesis was planned but was not performed because the volume of drainable fluid decreased substantially after diuresis; therefore, the serum-ascites albumin gradient (SAAG) and ascitic total protein concentration were unavailable. When ascitic fluid can be obtained, cardiac ascites classically demonstrates a SAAG ≥1.1 g/dL with a total protein concentration >2.5 g/dL, helping distinguish it from cirrhotic ascites, which typically has lower ascitic protein [5]. In this case, the rapid reduction in ascites with decongestion provided additional support for a cardiac mechanism.

This case also highlights the importance of hemodynamic assessment in patients with suspected cardiohepatic syndrome. In our patient, left heart catheterization demonstrated a markedly elevated left ventricular end-diastolic pressure. Right heart catheterization can further provide valuable information regarding right atrial pressure, pulmonary artery pressures, pulmonary capillary wedge pressure, cardiac output, and response to diuretic therapy [6]. In patients with persistent congestion or unclear volume status, hemodynamic assessment may help guide management and clarify the level to which elevated filling pressures impact hepatic dysfunction and ascites [6].

Although pulmonary congestion is the most recognized manifestation of acute decompensated heart failure, persistent elevation of right-sided filling pressures can also result in clinically significant systemic venous congestion involving the liver, kidneys, gastrointestinal tract, and serosal spaces. These manifestations, including congestive hepatopathy, ascites, pleural effusions, hyponatremia, and cardiorenal syndrome, should remain important diagnostic considerations, particularly in patients with preserved left ventricular systolic function, and warrant an assessment of volume status and right-sided hemodynamics [2].

In addition, the degree of concentric left ventricular hypertrophy despite reportedly controlled hypertension prompted concern for restrictive or infiltrative cardiomyopathy. Severe systemic congestion, preserved ejection fraction, elevated filling pressures, and disproportionate ventricular hypertrophy suggested possible underlying restrictive physiology, including infiltrative disorders such as cardiac amyloidosis. Further outpatient evaluation with cardiac MRI was therefore recommended. 

During this admission, management appropriately focused on decongestion and guideline-directed therapy for HFpEF, including intravenous furosemide, empagliflozin, and spironolactone [1]. Because cardiac amyloidosis remained a diagnostic consideration rather than a confirmed diagnosis, extending the discussion to disease-specific amyloidosis treatment would be premature. Outpatient cardiac MRI was recommended to clarify the underlying myocardial phenotype before considering disease-specific management.

What distinguishes this case is the convergence of marked systemic congestion, cholestatic liver injury, large-volume ascites, mesenteric congestion, pleural effusion, hypervolemic hyponatremia, and cardiorenal dysfunction despite preserved left ventricular ejection fraction and nonobstructive coronary artery disease. The substantial reduction in ascites and improvement in renal and hepatic abnormalities with decongestion further support a cardiohepatic mechanism. This case therefore illustrates how severe extracardiac congestion may dominate the presentation of HFpEF and mimic primary hepatobiliary disease, even in a relatively young patient.

Overall, this case demonstrates that preserved systolic function does not exclude severe hemodynamic compromise or extensive extracardiac manifestations of heart failure. Recognition of congestive hepatopathy and cardiac ascites is essential to avoid unnecessary hepatobiliary interventions and to appropriately direct treatment toward aggressive decongestion and optimization of underlying cardiac pathology [7].

This report has several limitations. Diagnostic paracentesis could not be completed after the ascites diminished with diuresis; therefore, SAAG and ascitic total protein values were unavailable. Right heart catheterization was not performed, and direct right-sided hemodynamic confirmation was unavailable. Results of the recommended outpatient cardiac MRI and subsequent evaluation for infiltrative cardiomyopathy were also unavailable. Patient-specific information regarding medication access, housing stability, food insecurity, transportation, and other social factors that might have influenced heart-failure management or follow-up was not available. Accordingly, the diagnoses of congestive hepatopathy and cardiac ascites were based on the combined clinical, biochemical, imaging, left-sided hemodynamic, and treatment-response findings rather than on ascitic-fluid analysis or direct right-sided hemodynamic confirmation.

## Conclusions

Congestive hepatopathy and cardiac ascites are important but underdiagnosed manifestations of systemic venous congestion in HFpEF. Patients with preserved ejection fraction may still develop severe congestion related to elevated filling pressures, with manifestations such as liver injury, ascites, pleural effusions, hyponatremia, and cardiorenal syndrome. Recognition of the cardiohepatic relationship remains important because these findings may mimic primary hepatobiliary disease. As such, early identification, along with timely and appropriate hemodynamic management, may improve outcomes while helping to prevent progression toward chronic hepatic fibrosis and cardiac cirrhosis. Further investigation is needed to better define the prevalence of congestive hepatopathy in patients with HFpEF and determine whether earlier recognition and targeted decongestive therapy can improve long-term hepatic and cardiovascular outcomes.
